# Well Water Arsenic Exposure, Arsenic Induced Skin-Lesions and Self-Reported Morbidity in Inner Mongolia

**DOI:** 10.3390/ijerph6031010

**Published:** 2009-03-09

**Authors:** Yajuan Xia, Timothy J. Wade, Kegong Wu, Yanhong Li, Zhixiong Ning, X Chris Le, Xingzhou He, Binfei Chen, Yong Feng, Judy L. Mumford

**Affiliations:** 1 Inner Mongolia Center for Endemic Disease Control and Research, Huhhot, Inner Mongolia, China; E-Mails: yajxia@sohu.com (Y.X.); wkg1959@163.com (K.W.); nmgliyh@163.com (Y.L.); 2 U.S. Environmental Protection Agency National Health and Environmental Effects Research Laboratory, Chapel Hill, North Carolina, USA; E-Mails: wade.tim@epa.gov (T.W.); mumford.judy@epa.gov (J.M.); 3 Ba Men Anti Epidemic Station, Bayingnormen, Inner Mongolia, China; E-Mail: bsjkxd@163.com; 4 University of Alberta, Department of Public Health Sciences, Edmonton, Canada; E-Mail: xc.le@ualberta.ca; 5 Chinese Academy of Preventative Medicine, Institute of Environmental Health & Engineering, Beijing, China; E-Mail: He 2007@188.com; 6 Computer Sciences Corporation, RTP, North Carolina; E-Mail: chen.binfei@epa.gov; 7 Hangjin Hou Centers for Disease Control, Inner Mongolia, China; E-Mail: Bingyu33@sina.com

**Keywords:** Arsenic, drinking water, Inner Mongolia, hyperkeratosis, skin lesions

## Abstract

Residents of the Bayingnormen region of Inner Mongolia have been exposed to arsenic-contaminated well water for over 20 years, but relatively few studies have investigated health effects in this region. We surveyed one village to document exposure to arsenic and assess the prevalence of arsenic-associated skin lesions and self-reported morbidity. Five-percent (632) of the 12,334 residents surveyed had skin lesions characteristics of arsenic exposure. Skin lesions were strongly associated with well water arsenic and there was an elevated prevalence among residents with water arsenic exposures as low as 5 μg/L-10 μg/L. The presence of skin lesions was also associated with self-reported cardiovascular disease.

## 1. Introduction

Chronic arsenic exposure from contaminated well water has been associated with cancer and non-cancer health effects including bladder and lung cancer, vascular and cardiovascular effects, diabetes, peripheral neuropathies, and adverse birth outcomes [1–10]. In the Bayingnormen (Ba Men) region, located on the Hetao Plain, north of the Huang He (Yellow) River of western Inner Mongolia (P.R. China), arsenic is naturally occurring in ground water, especially in the three counties of Hangjin Hou, Lin He and Wu Yuan. In the late 1970s, the primary source of drinking water shifted away from large shallow wells to deeper artesian wells, which were often contaminated by naturally occurring arsenic. Skin lesions characteristic of arsenic exposure were reported in the region in the early 1990s. Such skin lesions are usually manifested as hyperpigmentation on the trunk or extremities, and hyperkeratoses on the palms and feet and may be associated with future cancer risk [11].

**Figure 1.**
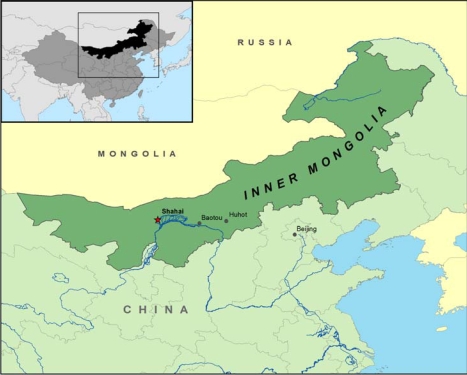
Study site and vicinity.

We conducted a census of one arsenic-exposed village in the Ba Men region, Shahai village located in Hangjin Hou county (Figure 1) with the goal of documenting arsenic exposures in the region, recording the prevalence of arsenic induced skin lesions, and describing the demographic factors associated with arsenic exposures, and arsenic toxicity.

## 2. Methods

### 2.1. Census

Interviewers from the Inner Mongolia Center for Endemic Disease Control and Research (IMCEDCR), and the Ba Men Anti Epidemic Station visited each family in the village and interviewed an adult household member during November and December 2004. Two interviewers visited each household, and at least one was a medical professional (nurse or physician). The interviews included questions about demographic characteristics for each person living in the household since January 1, 1997, including date of birth, sex, ethnicity, occupation and total household income. The respondent was also asked whether each family member had ever been diagnosed with diabetes, stroke, cardiovascular disease, cancer (location and type), or arsenic-associated skin lesions (hyperpigmentation, hyperkeratosis or depigmentation) and when these conditions had been first diagnosed. Cardiovascular disease was defined as any history of the following conditions: ischemic heart disease, hypotension, hypertension, congestive heart failure, and cardiac arrhythmias. Only those illnesses which were diagnosed by a doctor were considered. Whenever possible, the medical member of the interview team assessed the presence of these skin lesions by visual examination The field team included medical outreach workers from the Inner Mongolia Centers for Endemic Disease Control and Research and the Ba-Men Anti-Epidemic Station. These researchers have many years of experience in determining the presence of skin lesions and have reported their presence in previous published work [11–13]. Because most residents were farmers, or otherwise did not work out of the home, and interviews were conducted during the winter when the families were at home, the vast majority (at least 90%) of reported skin lesions were able to be confirmed by visual inspection. Interviews also included information regarding smoking habits (current smoker, quit, and years smoked), alcohol use (every day, sometimes or never) and a detailed residence history. The residence history consisted of a description of the water source (hand pump well, large mouth well, piped water from a community well, or other), and the dates and location of the residence for each family member for the past 30 years.

Research staff at the Ba Men Anti-Epidemic Station double-entered all data in a MS Access database. Discrepancies were identified and corrected by referring to the original survey form and if necessary, contacting the respondent for additional information and clarification.

### 2.2. Water Collection, Arsenic Analysis and Exposure Assignment

At the time of interview, we collected approximately 40 mL of water in 50 mL polypropylene test tubes at the household’s primary source of water. After collection, the samples were stored at −20º C and then delivered on ice via air to University of Alberta in Edmonton, Canada for analysis. We measured total arsenic in water samples using inductively coupled plasma mass spectrometry (ICPMS) as described previously [13,14]. The detection limit for ICPMS is 0.1 μg/L. For some households (N=141), water samples were analyzed by the IMCEDCR using Atomic Fluorescence (AF, detection limit=0.4 μg/L).

We assigned arsenic exposures based on the measured arsenic value from the resident’s well, shared well, or community well. For those results below the detection limit, we assigned one-half the detection limit as the exposure value.

### 2.3. Statistical Analysis

We used simple frequency tables to describe demographic and baseline characteristics of the cohort. We used bivariate tabulations (cross-tabulations) to examine differences in the prevalence of reported health conditions, smoking, age, and other factors by sex and mortality. To evaluate group differences by arsenic exposure, we used t-tests and one-way analysis of variance.

To analyze self-reported morbidity in relation to arsenic exposure, we estimated the odds of illness as a function of arsenic exposure using logistic regression models. We also considered self-reported skin lesions as a marker of chronic arsenic exposure and used logistic regression models to evaluate the prevalence of these skin lesions and self-reported morbidity for the other conditions. To account for potential biases in exposure assessment resulting from residents who recently changed their water source, we separately analyzed residents who reported using their same well since before 1995 (approximately 10 years) and since before 1985 (approximately 20 years).

In the regression analyses, we controlled for age in years (continuous), sex (male versus female), education level (coded as an ordinal variable, 1 through 5, ranging from incomplete primary school through college degree), smoking, household income, water source type (hand pump well or other), and current alcohol consumption (yes or no). We categorized smoking status as never smoked, smoked under a year, smoked 1 to 10 years, smoked 11–20 years, or smoked over 20 years. Because household income was missing for approximately 5% of the sample, these missing values were imputed by best subset regression [15] using available characteristics such as household size, education levels, occupation type, water source type, and other demographic characteristics. We compared the change in the arsenic coefficient from the fully adjusted models, to models adjusting for just age and sex (reduced model), and an unadjusted model. Because the fully adjusted model usually indicated at least some confounding (based on a change in the coefficient of the arsenic among the models), we reported results of the fully adjusted models. We used Stata 9.2 [16] for all statistical analysis.

## 3. Results

### 3.1. Cohort Description

We interviewed a total of 3,284 households and collected information about 12,334 individuals. Of these 11,416 had complete information for all key covariates and outcomes. No households refused to participate and at least one member of all households in the village was interviewed. Demographic characteristics of the residents are shown in Table 1. The majority of residents were farmers (60%), and were from the Han ethnic group (99%). Overall, 31% had smoked at some point in their life (ever smokers), but this was heavily imbalanced by sex: fifty-six percent of men were ever smokers, compared to only 6% of women. Women were also more likely than men to have not completed at least primary school (27% of women and 14% of men did not complete primary school), and less likely to drink regularly (2% of women and 44% of men reported drinking at least some alcohol).

### 3.2. Arsenic Exposure

Seventy-one percent of respondents reported using a hand-pump well as their current source of water (Table 1). Although local health officials have recently been working to provide new water sources to residents in areas affected by arsenic, residents reported using their current water source a mean of 9.9 years. Arsenic measures were not available for 52 households due to a mislabeled, damaged, lost sample or insufficient sample volume. An additional 141 households were assigned arsenic values using data provided by the IMCEDCR. Total arsenic in water samples from current primary source of water ranged from below detection to 637.7 μg/L, with mean exposure of 37.9 μg/L (median 21 μg/L). A total of 65 households had results for water arsenic below the detection limit (46 with the ICPMS method and 19 from the AF method) and were assigned arsenic exposures one-half the detection limit. Average arsenic exposure did not differ by smoking (p=0.78), ten-year age category (p=0.45), alcohol use (p=0.30), or occupation (p=0.20). Lower completed education level was associated with higher levels of arsenic exposure (p=0.001) as was lower household income (p=0.04). Those who completed a college degree had significantly lower average arsenic exposure (27.8 μg/L) compared to those with middle school (p=0.025), primary school (p=0.01) or less than primary (p=0.002) school education (37.6, 38.5 and 40.7 μg/L, respectively). Residents using hand pump wells as their primary water source had higher average arsenic exposure compared to residents with community wells or other water sources (46.0 μg/L and 18.3 μg/L, respectively, p<0.0005), although the other types of water sources (including community wells), also had high levels of arsenic with exposures up to 138 μg/L. Arsenic measures were available from 57 hand-pump wells in the current study were also previously measured by the Ba Men Anti-Epidemic Station in 1992 [17]. The arsenic levels from these wells measured in 1992 showed a good correlation to these same wells measured during this study (Pearson’s r=0.76, p<0.0005).

### 3.2. Skin Lesions

Over 5% (632 individuals) had skin lesions (hyperkeratosis, hyperpigmentation, depigmentation) characteristic of arsenic exposure. Those with skin lesions had higher average arsenic exposure levels compared to those without skin lesions (61.6 μg/L and 36.9 μg/L, respectively, p<0.01). Demographic characteristics and the presence of such skin lesions are shown in Table 2. Skin lesions were observed in children as young as 11 years, and prevalence peaked at approximately 11% among those aged 41–60. After adjustment in a multivariate logistic regression model, female gender, alcohol use, farm work, education level and water source were associated with skin lesion prevalence. Residents who did not complete primary school had the highest skin lesion prevalence (8%), which decreased as education level increased to a prevalence of only 1.6% among those with a college degree. Presence of skin lesions was also strongly associated with farm work (8% among farmers; 1% among other occupations, p<0.01), and alcohol use (p<0.01). Those with water piped from community wells had a higher prevalence of skin lesions, compared to those using hand-pump wells, but these wells, as noted above, also had high levels of arsenic exposure.

The presence of skin lesions was strongly associated arsenic exposure in a dose-dependent manner. Under 2% of those exposed to levels of 5 μg/L and under had skin lesions compared to approximately 10% of those exposed to arsenic levels 100 μg/L or greater (Table 2). Each 50 μg/L increase in arsenic exposure was associated with a 35% increase in the odds of skin lesions (OR=1.35, p<0.01) with a stronger trend evident among males (OR=1.43, p<0.01 and OR=1.27, p<0.01 among males and females, respectively). Those exposed to arsenic between 5–10 μg/L had 2.5 times odds of skin lesions compared to those exposed to arsenic under 5 μg/L (OR=2.52, p<0.01, Tables 2, 3). Those exposed to arsenic between 100 and 300 μg/L had nearly 9 times greater odds of skin lesions (OR=8.83, p<0.01, Tables 2, 3). A similar relationship was also evident among residents exposed at least 10 years (since before 1995), exposed at least 20 years (since before 1985) and among only those households with a hand pump well, with significantly elevated risks for skin lesions apparent among residents exposed to wells with arsenic levels of 5–10 μg/L and 10–20 μg/L (Table 3).

## 3.3. Self-Reported Disease

Due to the low prevalence of diabetes (44 cases, <1% prevalence) and cancer (11 cases, <1% prevalence) we concluded the sensitivity to be too low to be accurately evaluated with regard to arsenic exposure or skin lesion occurrence. These conditions may not have prompted a hospital visit and doctor diagnosis. Self reported, doctor diagnosed, cardiovascular disease was reported by 988 individuals (8%) and stroke by 127 (1%). Stroke was not associated with arsenic exposure (OR=1.03, p=0.59 per 50 μg/L increase). Cardiovascular disease showed a borderline association with arsenic exposure among males (OR=1.10, p=0.07 per 50 μg/L increase) but a trend was not evident among females (OR=0.99, p=0.80). Among all subjects, 12% (12/92) of the most highly exposed individuals (>300 μg/L) reported cardiovascular disease compared to only 8% exposed to arsenic under 5 μg/L, but following adjustment for covariates this differences was not statistically significant (OR=1.72, p=0.16). In the multivariate model, ever smoking was a significant risk factor for both stroke and cardiovascular disease (OR=1.92, p=0.01 and OR=1.47, p<0.01 comparing ever smokers to never smokers for stroke and cardiovascular disease, respectively). The presence of skin lesions was associated with self reported cardiovascular disease (OR=1.62, p<0.01), but not stroke (OR=1.04, p=0.89)

## 4. Discussion

Since the 1980s, residents of the Ba Men region of rural Inner Mongolia have been chronically exposed to low to moderate levels of arsenic. In comparison with highly exposed populations in West Bengal, India [18] Bangladesh [19], Taiwan [3], Chile [8], and Argentina [5], arsenic exposures in Shahai village were relatively low. Fewer than 1% of residents in Shahai Village were exposed to arsenic levels more than 300 μg/L, and slightly over 5% of residents had skin lesions. Guo [20] observed a prevalence of skin lesions in 44% respondents in a study conducted in a nearby part of Inner Mongolia, however up to 92% of residents were exposed to arsenic levels above 50 μg/L compared to only 13% of residents in Shahai village. We observed an elevated prevalence of skin lesions among those with well water arsenic between 5 and 10 μg/L, suggesting that arsenic induced toxicity may occur at these relatively low exposures with chronic exposure. Since we did not have detailed information on arsenic exposures at previous residences or water sources, it is possible this result could have reflected recent exposures which were not representative of past exposures. However, we found the elevated risk for those exposed between 5 and 10 μg/L remained even among those using the same well since before 1995 and since before 1985. Since current users of hand pump wells are more likely to rely only on a single well and therefore may have had a more accurate exposure assessment, we analyzed these residents separately and observed a similar relationship at low exposures (Table 3). Others have reported skin lesions at exposures near or below 100 μg/L [18,21]. Recent studies in Bangladesh reported elevated skin lesions at levels as low as 8.1–40 μg/L [22] and a second study reported slightly elevated risks at exposures as low as 11–50 μg/L with clear elevated risks occurring at exposures in excess of 50 μg/L [23].

Arsenic exposure has been suggested to be a necessary, but not a sufficient cause of skin lesions [24]. Several studies have observed skin lesions at relatively low levels of exposure (less than 50 or less than 100 μg/L), and there is increasing evidence that cofactors or co-exposures may act together with arsenic exposure to promote the development of such lesions. In our study, socioeconomic factors such as education level and income were associated with both arsenic levels and arsenic induced skin lesions. The presence of skin lesions was associated with lower education, community well use, farm work and alcohol use. Because education level and income were also associated with arsenic levels these socioeconomic factors probably indicate a disparity in exposure to arsenic throughout the study area, although these factors remained associated with skin lesions after controlling for arsenic exposure. Age, sex, education and household income were independently associated with chronic arsenic poisoning in Bangladesh [25]. Another recent study in Bangladesh also observed a positive interaction between SES (in particular, land ownership) and the effect of arsenic on skin lesion risk [26].

Although women had a slightly higher overall prevalence of skin lesions, our results suggest some susceptibility among men to skin lesions following arsenic exposure. This susceptibility among males is consistent with results reported from Bangladesh [22,27]. Other studies have also reported differences in susceptibility in skin lesions with arsenic exposure by gender [22,28–31], smoking [24] and sunlight exposure [24]. We did not observe a significant interaction effect by smoking status, but because most smokers were males and few females smoked, it was difficult to distinguish these effects reliably. Reasons for differences in susceptibility are not known, but differences in nutrition [32–34], methylation capacity[35], sunlight exposure and tobacco use and use of fertilizers [24] and genetic susceptibility resulting from variations in oxidative stress genes [36], differences in DNA repair capacity[37,38] polymorphisms in GST genes affecting the behavior of Glutathione *S*-transferases [39] or susceptibility to genetic damage [40] have been suggested as possible cofactors.

Although well water arsenic exposures were not strongly associated with self reported disease (with the exception of skin lesions), those with skin lesions were also more likely to report being diagnosed with cardiovascular disease a condition that has previously been associated with arsenic exposure [7,10,41]. Other studies have also observed increased morbidity including peripheral neuropathy and respiratory illness among residents with skin lesions compared to those without [42].

It is likely at least some of the self-reported conditions were under reported or incorrectly reported, and we found the very low prevalence of self-reported diabetes and cancer to be unreliable for analysis. However, the prevalence of self-reported, doctor-diagnosed cardiovascular disease and stroke were broadly consistent with prevalence reported by a similar method of assessing prevalence in the United States [43,44] and showed an association with ever smoking, a known risk factor these conditions. These conditions may be more likely to prompt a doctor’s visit and a diagnosis, making their self-reported prevalence more reliable. We do not expect there to have been significant systematic bias associated with misclassifications resulting from self-report. Because residents were not made aware of the arsenic levels in their water any effects of misclassification would be random and tend reduce the associations toward a null effect. Despite these limitations, a major strength of the study was our ability to visit and interview and obtain health and exposure information from all households in the village. Additional misclassification may have occurred by relying on the arsenic levels measured from the current water-source. The exposure misclassification associated with these limitations, however, is minimized since the vast majority of residents in this region rely solely on their household well for their water. As most of the residents are farmers, they do not work outside of the home and therefore have limited access to other water sources. Based on our comparison with arsenic measures of the 57 wells for which information was available in 1992, we were able to provide some evidence of the stability of arsenic in these wells over time.

It is unlikely that exposure to other sources of arsenic or other water sources affected our study results. Guo [45] reported low levels of arsenic in the soil and crops of arsenic affected villages in Inner Mongolia. There is no evidence of other major sources of arsenic exposure, for example, from indoor coal combustion or from pesticide applications [13] and previous studies have reported that drinking water is the only significant source of arsenic contamination [46].

We conducted several sensitivity analyses to confirm our approach to analysis and interpretation was robust. We confirmed that using the original household income rather than the imputed values did not change the results substantially, but by using imputed values, we were able to allow additional observations to be considered in multivariate models. For arsenic values below the limit of detection, we assigned one-half the detection limit. Assigning other nominal values (zero, or the detection limit) had no affect on the results since the categorization remained the same. Excluding these values only affected the point estimates only very slightly but resulted in the reduction of 252 individual observations. Since some of the outcomes such as cardiovascular disease are diseases of older adults, we also confirmed that excluding children did not affect the result or the interpretation.

Residents of Shahai village have been chronically exposed to moderate levels of arsenic from well water for 20 years. Increased prevalence of skin lesions characteristic of chronic arsenic exposure were observed among residents with arsenic measures in well water as low as 5–10 μg/L. Skin lesions were also associated with self-reported prevalence of cardiovascular disease.

## Figures and Tables

**Table 1.** Characteristics of residents of Shahai village, Inner Mongolia ^a^.

	Number	Per cent
**Sex**		
Male	6 202	50.4%
Female	6 107	49.6%
**Age category**		
0–5	457	3.7%
6–10	628	5.1%
11–15	1 195	9.7%
16–20	1 311	10.6%
21–30	2 033	16.50%
31–40	2 315	18.8%
41–50	1 813	14.7%
51–60	1 331	10.8%
61–70	841	6.8%
71–80	337	2.7%
>80	61	0.5%
**Occupation**		
Agriculture	7 404	60.1%
Industry	300	2.4%
Professional	81	0.7%
Teacher	169	1.4%
Student	2 325	18.9%
Service	750	6.1%
Not employed	899	7.3%
Other	401	3.3%
**Smoking**		
Never smoker	8,475	69.0%
Smoked 1 year or less	73	0.6%
Smoked 1–10 years	1,132	9.2%
Smoked 10–20 years	1,164	9.50%
Smoked over 20 years	1,437	11.7%
**Education**		
College	341	2.8%
high school	1 241	10.1%
middle school	5 073	41.2%
Primary school	3 120	25.3%
some primary or none	2 536	20.6%
**Frequency of alcohol use**		
every day	99	0.8%
sometimes	2 701	22.0%
Never	9 492	77.2%
**Household income (Yuan, imputed)^b^**		
0–999	246	2.0%
1,000–9,999	8,407	68.2%
>=10,000	3,681	29.8%
**Arsenic exposure**		
0–5	3,467	28.5%
5.1–10	900	7.4%
10.1–20	1,336	11.0%
20.1–50	3,670	30.2%
50.1–100	1,624	13.4%
100.1–300	1,072	8.8%
>300	95	0.8%
Mean=37.94 μg/L Min=0.05 μg/L 25^th^ percentile= 3.4 μg/L 75^th^ percentile=43.6 μg/L Max=637.7 μg/L		
**Water source**		
Hand pump well	8 726	71.0%
Community well	3 552	28.9%
Other	13	0.1%

a) Categories do not add to 12,894 due to missing and unknown responses.

b) HH income category imputed for missing responses using best-subset regression, see discussion in methods section.

**Table 2.** Associations between demographic characteristics and skin lesions (N=11,416)^a^.

	Skin lesions present		Odds Ratio 95 % CI p-value
	No.	%	
**Sex**			
male	293	5.10%	Ref
female	329	5.80%	1.86 1.45, 2.37 <0.01
**Age category**			
0–10	0	0.00%	
11–15	7	0.60%	
16–20	10	0.90%	
21–30	37	2.00%	
31–40	124	5.70%	
41–50	181	10.60%	
51–60	139	11.10%	
61–70	90	11.40%	
71–80	31	9.80%	
>80	3	5.40%	1.04^b^ 1.03,1.04 <0.01
**Education**			
college	5	1.70%	
high school	41	3.60%	
middle school	224	4.80%	
primary school	157	5.40%	
some primary or none	195	8.10%	0.87^c^ 0.78,0.97 0.02
**Drinking**			
never drinker	411	4.70%	Ref
ever drinker	211	8.10%	1.59 1.24,2.04 <0.01
**Farm worker**			
No	51	1.10%	Ref
Yes	571	8.20%	3.93 2.86,5.41 <0.01
**Smoking**			
never smoker	341	4.30%	Ref
smoked 1 year or less	2	3.00%	0.95 0.25,3.69 0.95
smoked 1–10 years	42	4.00%	0.92 0.63,1.33 0.66
smoked 10–20 years	83	7.60%	1.11 0.80,1.52 0.54
smoked over 20 years	154	11.30%	1.33 0.98,1.81 0.07
**Arsenic categories**			
0–5 μg/L	58	1.80%	
5.1–10 μg/L	32	3.80%	2.52 1.47,4.30 <0.01
10.1–20 μg/L	53	4.20%	2.83 1.77,4.53 <0.01
20.1–50 μg/L	235	6.90%	3.94 2.78,5.59 <0.01
			6.03 4.05,8.97 <0.01
50.1–100 μg/L	128	8.30%	
100.1–300 μg/L	107	10.50%	8.83 5.77,13.51 <0.01
>300 μg/L	9	9.80%	7.94 2.73,23.12 <0.01
**Yearly HH Income(Yuan, imputed)**			
0–999	15	6.50%	
1000–9999	437	5.60%	
>=10000	170	5.00%	1.13^c^ 0.93,1.37 0.22
**Water source**			
Other^d^	213	6.60%	
Hand pump well	409	5.00%	0.52 0.41,0.65 <0.01

a) Table only considers 11,416 respondents with complete data.

b) Odds ratio per year increase in age.

c) Odds ratio for trend in increasing education and increasing income category.

d) Consists of residents with a community well (99%) and unknown water source (1%).

**Table 3:** Adjusted odds ratios for skin lesions by category of arsenic exposure^a^.

Arsenic exposure	All Subjects	Residents exposed since before 1995	Residents exposed since before 1985	Hand pump well users
	Odds Ratio 95% CI P-value Number	Odds Ratio 95% CI P-value Number	Odds Ratio 95% CI P-value Number	Odds Ratio 95% CI P-value Number
0–5 μg/L	Ref 3 215	Ref 1 678	Ref 477	Ref 2 318
5.1–10 μg/L	2.52 1.47,4.30 <0.01 845	2.34 1.21,4.51 0.01 411	2.08 0.70,6.20 0.19 128	1.90 1.08–3.36 0.03 727
10.1–20 μg/L	2.83 1.773,4.525 <0.01 1 277	2.45 1.35,4.44 <0.01 663	3.18 1.36,7.45 <0.01 235	2.12 1.28–3.54 <0.01 1 080
20.1–50 μg/L	3.94 2.78,5.59 <0.01 3 429	3.31 2.20,4.98 <0.01 1 862	2.44 1.10,5.43 0.03 316	2.83 1.84–4.35 <0.01 1 558
50.1–100 μg/L	6.03 4.05,8.97 <0.01 1 537	5.38 3.31,8.75 <0.01 667	3.62 1.57,8.33 0.003 226	4.01 2.62–6.14 <0.01 1 381
100.1–300 μg/L	8.83 5.77,13.51 <0.01 1 021	10.38 6.06,17.77 <0.01 407	8.99 4.15,19.46 <0.01 155	6.59 4.28–10.11 <0.01 1 013
>300 μg/L	7.94 2.73,23.12 <0.01 92	12.64 3.209,49.822 <0.01 43	N/A^b^	5.92 2.04–17.17 <0.01 92

a) Adjusted odds ratios estimated from logistic regression, controlling for drinking, smoking, education, sex, farm work, income, well type and age.

b) Top two categories combined due to few numbers exposed over 300 μg/L.
